# Kikuchi-Fujimoto Disease With Encephalopathy in Children: Case Reports and Literature Review

**DOI:** 10.3389/fped.2021.727411

**Published:** 2021-10-01

**Authors:** Yu-Ting Pan, Li-Ming Cao, Yan Xu, Zhi-Dan Fan, Hai-Guo Yu

**Affiliations:** ^1^Department of Rheumatology and Immunology, Children's Hospital of Nanjing Medical University, Nanjing, China; ^2^Department of Infectious Disease, Children's Hospital of Nanjing Medical University, Nanjing, China

**Keywords:** encephalopathy, Kikuchi-Fujimoto disease, lymphadenopathy, meningitis, glucocorticoids

## Abstract

**Background:** Kikuchi-Fujimoto disease (KFD) is a benign and self-limiting disease characterized by regional lymphadenitis and low-grade fever. Encephalopathy may present in children with KFD. We present three cases of KFD with encephalopathy in children and a literature review.

**Methods:** Literature published between 2010 and 2020 was reviewed to understand the clinical features, laboratory findings, and treatments for encephalopathy occurring in children with KFD.

**Results:** The interval between KFD and onset of neurological symptoms was 10 days to 3 months. Laboratory results were normal, except for high protein levels in cerebrospinal fluid findings. Brain magnetic resonance imaging (MRI) findings include hyperintense T2 and FLAIR signal in the supratentorial white matter, deep gray matter, brain stem, cerebellum, temporal lobes, pons, and basal ganglia. Glucocorticoids and immunoglobulin could be effective for treating KFD with encephalopathy.

**Conclusion:** The early clinical manifestations of KFD with encephalopathy in children lack specificity, and the diagnosis is mainly based on CSF analysis and brain MRI findings. Early and timely immunomodulatory therapy is effective and can improve the prognosis of patients with KFD with encephalopathy.

## Introduction

Kikuchi-Fujimoto disease (KFD), also termed subacute necrotizing lymphadenitis or histiocytic necrotizing lymphadenitis, was first described in Japan in 1972 (1, 2). KFD is an important cause of lymphadenitis in children. The pathology and etiology remain uncertain, particularly regarding infectious causes (3). The onset is usually acute or subacute. Clinical manifestations include cervical lymph node enlargement with low-grade fever and leukocytopenia in a previously healthy child (4). Differential diagnoses include malignant lymphomas or infections (5). Differentiation between lymphadenopathy in systemic lupus erythematosus (SLE) and KFD is difficult because KFD can precede, postdate, or coincide with SLE (6). Lymph node biopsy remains the gold standard for the diagnosis of KFD (7). Severe primary central nervous system (CNS) involvement, such as aseptic meningitis and meningoencepathitis (ME), can be rare complications of KFD in children (8). These complications can present as headache, peripheral neuropathy, tonic spasms, and convulsions (9). Recognition of CNS involvement associated with KFD may help evaluate patients presenting with encephalopathy and regional lymphadenopathy. It is imperative to understand the neurological complications in KFD to avoid unnecessary treatment. We report three cases of Chinese children with KFD with concomitant encephalopathy. We also reviewed encephalopathy-related conditions observed in children with KFD.

## Case Presentation

### Case 1

**Figure 1:**
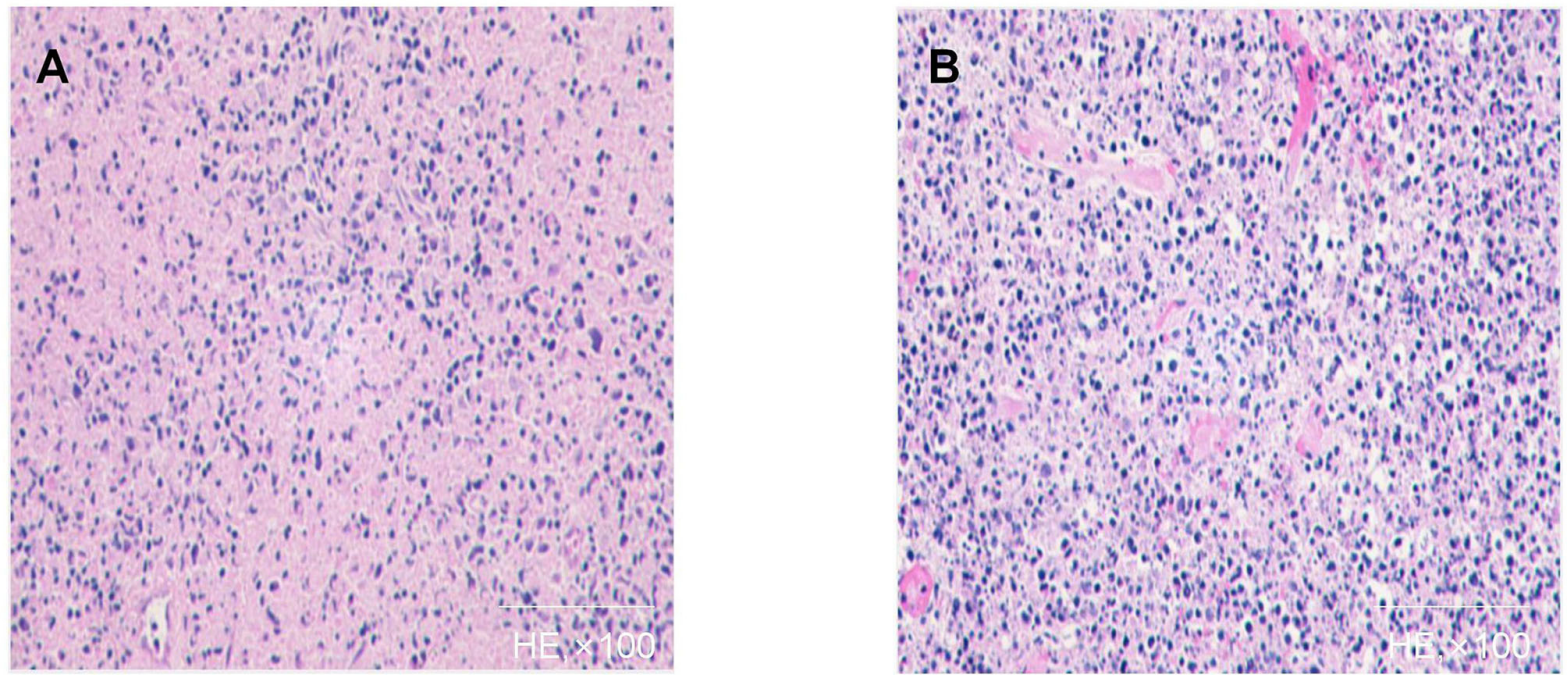
Histopathology of affected lymph node of case 1 **(A,B)**: Patchy circumscribed areas with eosinophilic fibrinoid necrosis in the paracortex and cortex and absence of granulocytes in the areas of necrosis are shown. A variety of cells surround the necrotic area, and a large number of apoptotic cell fragments exist outside the cells.

A 9-year-old boy presented with fever and malaise in September 2019. After 2 weeks, this was accompanied by a painful and tender left anterior cervical lymphadenopathy. He was diagnosed with bacterial lymphadenitis by a local physician and was prescribed ceftriaxone and methylprednisolone. Oral antipyretics did not provide relief. Ten days later, there was persistent low-grade fever (axillary temperature, ≥37.5°C) with gradual lymph node enlargement. No apparent recent weight loss was observed. This prompted admission on the 18th day of illness at our hospital. Travel and exposure history was unremarkable. Physical examination was normal except for a slightly tender, 2.0 × 1.0 cm left anterior cervical lymph node. Laboratory test results showed a normal C-reactive protein (CRP), white blood cell (WBC) count of 3,060/mm^3^ with a low absolute neutrophil count of 1,010/mm^3^, and an erythrocyte sedimentation rate (ESR) of 21 mm/h. T-spot examination and purified protein derivative were negative. Serologic tests were negative for Epstein-Barr virus (EBV), cytomegalovirus (CMV), and hepatitis B virus (HBV) infection. He underwent lymph node biopsy, which revealed a large classic necrotic focus and proliferation of surrounding tissue cells suggestive of KFD (Figure 1). Supportive therapy, including non-steroidal anti-inflammatory drugs (NSAIDs) and intravenous dexamethasone (10 mg), was administered. His symptoms gradually improved. He was subsequently discharged with naproxen and a tapering dose of prednisone. However, he was readmitted 10 days later due to fever recurrence. He was treated with intravenous pentahydrate cefazolin and continuous oral prednisone therapy. Improvement was noted until the 8th day of re-admission when he had a transient headache with nausea and vomiting. Neck stiffness and Kernig's sign were absent. Physical examinations and neurologic examinations did not reveal any other abnormalities. Cerebrospinal fluid (CSF) analysis showed lymphocytic predominant pleocytosis (100 mm^3^, 77% lymphocytes), proteins of 86 mg/dL, and glucose of 43.24 mg/dL. Cerebrospinal fluid studies were negative for viral, bacterial, or fungal infections. CSF culture was sterile, and PCR for viral agents was negative. Neuroimaging revealed prominent meningeal enhancement. Additional medications, including naproxen, meropenem, acyclovir, and mannitol, were administered to decrease intracranial pressure. After 10 days, his condition improved significantly, and CSF analysis was normal. Brain magnetic resonance imaging (MRI) was normal. The patient remained well on follow-up.

### Case 2

**Figure 2:**
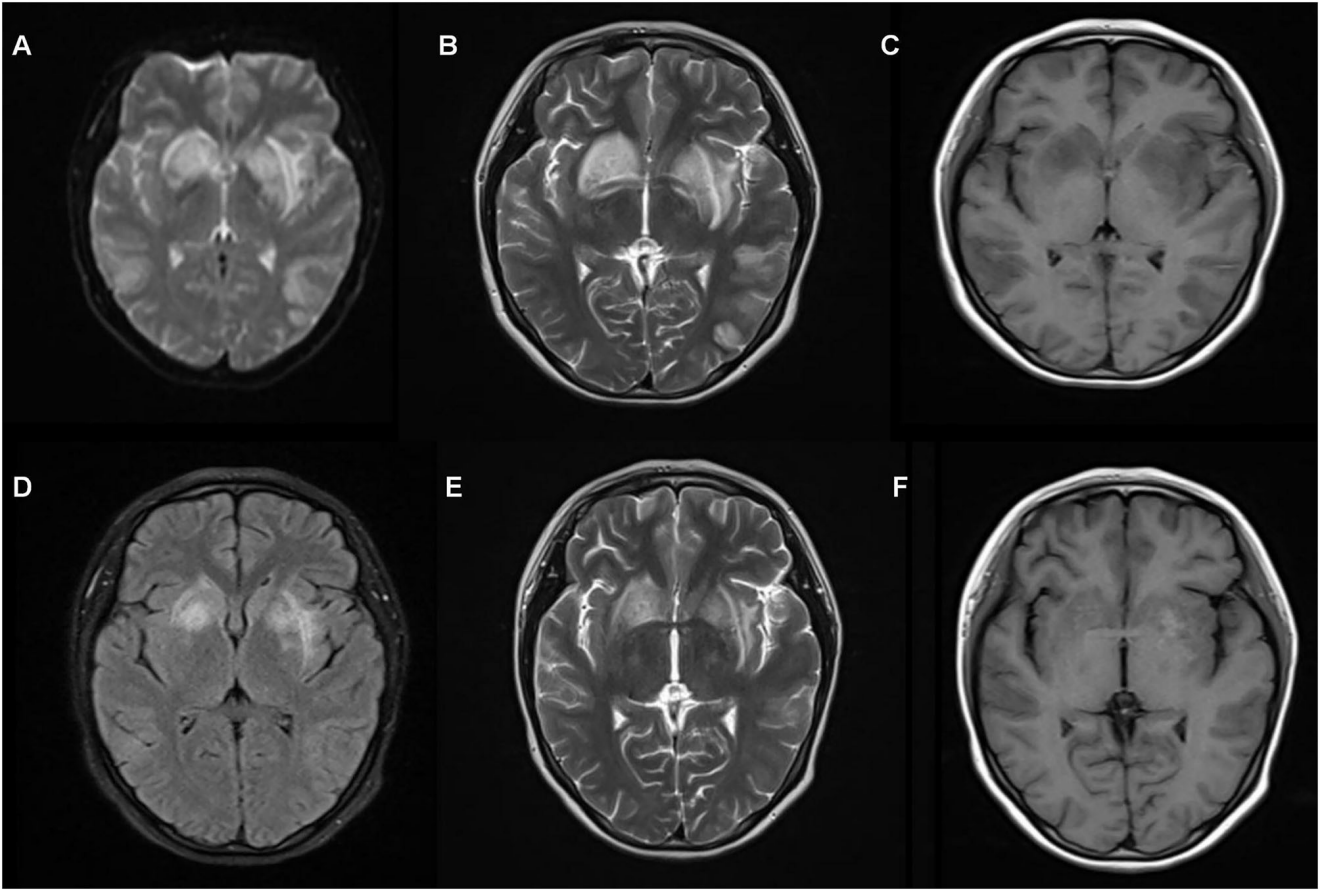
Magnetic resonance imaging (MRI) of case 2 **(A–C)** shows a wide range of abnormal signals in both hemispheres and basal ganglia region before pulse therapy. Follow-up brain MRI **(D–F)** after 15 days shows normal results.

A 15-year-old girl presented to our hospital with fever and tender swollen lymphadenopathy of 15 days duration. She was treated with antibiotics for 4 days without relief. Persistence of fever and lymphadenopathy prompted consult at our hospital. She had a history of KFD when she was 10 years old that presented similarly. She underwent lymph node excision and biopsy, which showed KFD. Prednisolone was administered and tapered over half a year. On physical examination, she had bilateral cervical lymphadenopathy, more prominent on the left side (2.5 × 2.5 cm), with multiple erythematous plaques on her cheek and chest. Laboratory results revealed mildly elevated inflammatory markers (CRP of 12 mg/L and ESR of 28 mm/h). Significant laboratory results were as follows: WBC, 2,330/mm^3^; lymphocyte count, 46.4%; neutrophil count, 1,040/mm^3^; and hemoglobin count, 12.1 g/dL. Tuberculin skin tests and blood cultures were negative. Rheumatologic markers (ferritin, anti-nuclear antibody, anti-neutrophil cytoplasmic antibody, anti-cyclic citrullinated peptide antibodies, extractable nuclear antigen antibody panel, anti-double stranded DNA, rheumatoid factor) were also negative. Lymph node biopsy revealed necrotic foci surrounded by macrophages (CD68+/MPO+) and plasmacytoid dendritic cells (CD68+/CD123+) consistent with KFD. Post-operatively, intravenous dexamethasone (10 mg) and naproxen were administered. Symptoms resolved over the subsequent 48 h. However, on the 3rd postoperative day, she had severe headache and generalized tonic-clonic seizure lasting for several minutes. No meningeal signs were noted. Antibiotic therapy was shifted to intravenous ceftriaxone with the addition of acyclovir. She later presented with head twitching and syncope. She was immediately started on intravenous methylprednisolone (60 mg) therapy. MRI showed abnormal signals in both cerebral hemispheres and the basal ganglia region (Figure 2). CSF analysis showed elevated total protein levels (11.2 mg/dL). Other CSF parameters were normal. The extended autoimmune screening test did not reveal autoimmune encephalitis. Considering encephalopathy-associated KFD, intravenous immunoglobulin (IVIG), and pulse methylprednisolone (1 g/d) for 3 days followed by standard doses of prednisone (60 mg/d) were administered to suppress the immune response. She improved and was later discharged on a tapering course of oral prednisone. Follow-up cranial MRI was normal (Figure 2). The patient remained neurologically stable for 2 years.

### Case 3

A 7-year-old girl presented to our hospital with fever and tender right cervical lymphadenopathy of 20-day duration. She underwent surgery for Pierre-Robin syndrome when she was 2 years old. She was noted to have a tender right cervical lymphadenopathy of >2 cm in diameter without hepatosplenomegaly. On admission, her CRP level was normal. The complete blood count revealed a WBC count of 4,410 cells/mm^3^ and an ESR of 92 mm/h. EBV, CMV, and HBV serum tests were negative. An excisional cervical lymph node biopsy was performed, which showed paracortical hyperplasia accompanied by a large number of immunoblastic macrocells revealing KFD. The patient was treated with NSAIDs. After 14 days, her fever resolved with regression of the noted lymphadenopathy. The inflammatory markers remain elevated. The patient was discharged with naproxen, hydroxychloroquine, and a tapering dose of prednisone. Four months later, she had a 30-day history of rapidly progressive hyposthenia, ataxic gait, and urinary retention. The patient was brought to our emergency department due to status epilepticus, which presented as generalized tonic-clonic seizures with unconsciousness, lock-jaw, and blank stare. Oxygen support was provided, and intravenous diazepam and midazolam were administered. Physical and neurological examinations revealed hyperextension of the limbs. No other abnormalities were noted. Head computed tomography on admission revealed decreased white matter density. CSF analysis revealed an elevated number of cells (42/mm^3^, 82% lymphocytes) and proteins (22.7 mg/dL). The CSF sample was submitted for bacteriologic and viral examination and evaluated with serological and pathological tests. Growth was not observed in the non-specific or mycotic cultures of the CSF sample. The patient was diagnosed with encephalopathy associated with KFD. IVIG (1 g/kg) and an intravenous bolus dose of methylprednisolone (360 mg/d for 3 days) were administered. Plasma exchange was performed two times. After 4 weeks of treatment, the child was still unable to speak or sit without support. MRI findings showed remaining symmetrical abnormal signals in the bilateral basal ganglia with no apparent changes. The prognosis was poor.

## Discussion and Conclusion

KFD is primarily characterized by regional lymphadenopathy predominantly affecting adolescents (10). KFD is a self-limiting disease that usually resolves within 4–6 months. KFD has a reported recurrence rate of 3–4% in adults (11), while studies in children with KFD have shown high recurrence rates of up to 42.4% (12). Similar to previous reports, our three cases had cervical lymph node enlargement and fever as the presenting symptoms. Its etiology remains unknown. Various viruses, including CMV, EBV, parvovirus B19, and human herpesvirus 6, have been implicated as causative agents (13). The medical history, physical examinations, and medical imaging should be efficiently employed to rule out other forms of lymphadenitis. KFD mimics other common or more serious conditions, such as lymphoma or SLE; therefore, early and accurate diagnosis of KFD is crucial for avoiding unnecessary treatment. Cervical lymphadenopathy can also be the initial manifestation of SLE (12–26%) (14). Lymph node biopsy is the gold standard for the diagnosis of KFD, where in SLE usually presents with hematoxylin bodies and prominent plasma cells, which are rare in KFD (15). In contrast to lymphoma, the affected lymph nodes in KFD are solid, movable, and painful. Other laboratory tests and lymph node biopsy can rule out other diseases presenting as lymphadenopathy, such as SLE and NHL. Treatment of KFD includes NSAIDs, corticosteroids, and immunosuppressants (7).

Neurological complications associated with KFD have rarely been reported since they were first described in 1999 (16). We searched the literature on KFD with encephalopathy in children published between 2010 and 2020 in PubMed using keywords including “Kikuchi-Fujimoto Disease,” “encephalitis,” “encephalopathy,” and “CNS” with inclusion criteria age ≤ 18 years (Table 1). In a retrospective review of 244 cases, the incidence of neurological complications was reported to be approximately 5% (4). Neurological involvement can present as aseptic meningitis, acute cerebellar ataxia, brachial neuritis, brainstem encephalitis, neuromyelitis optica, spectrum disorder, peripheral neuropathy, and secondary blepharospasm. Aseptic meningitis is the most commonly reported neurological complication in KFD (2.5–2.8%). It is suggested that primary infection with unknown pathogens can produce causative substances, which may bind to target organs of the CNS. Avkan-Oguz et al. (20) reported a case of acute disseminated encephalomyelitis (ADEM) following KFD. Autoimmune response or immune reconstitution may result in concomitant KFD and ADEM. Interestingly, two of our patients developed neurological symptoms within a month after the biopsy. We hypothesized that biopsy might be a precipitating factor for neurological complications.

**Table 1:** KFD with encephalopathy in children published between 2010 and 2020 in PubMed.

	**Gender/Age**	**Neuro-imaging findings**	**Interval between onset of lymphadenopathy and neurological manifestations**	**Neurological manifestations**	**Cerebrospinal fluid (CSF) analysis**	**Treatment**	**Outcome**
Case 1	M/9	Prominent meningeal enhancement	6 weeks(4 weeks after biopsy)	Headache, nausea and emesis	Lymphocytic dominant pleocytosis high protein and glucose	Oral PD, IV acyclovir	Good
Case2	F/15	Abnormal signals in both hemispheres and basal ganglia region	Half of a month(3 days after biopsy)	Severe headache and a generalized tonic-clonic seizure	High protein	IV IG,IV MP,IV PD	Good
Case3	F/7	Abnormal signals in the bilateral basal ganglia	3 months	Hyposthenia, ataxic gait and urinary retention followed by status epilepticus	Pleocytosis, high protein	IV IG,IV MP	Bad
Byun et al. (8)	M/12	Hyperintense signal in cerebellum and posterior aspect of bilateral occipital lobes	3 weeks	Severe headache, meningeal sign, seizure	Pleocytosis, high protein	IV Dex	Good
	M/17	Hyperintense signal in right cerebellum and posterior aspect of brainstem	2 week	Behavior and personality change	Pleocytosis, high protein	Oral PD	Good
	F/15	Unremarkable (on CT)	10 days	Stupor mentality	Pleocytosis, high protein	IV MP, IV acyclovir, IV IG	Good
Jasti et al. (17)	F/15	hyperintense signal in dorsal midbrain and dorsal pons	2 weeks	Drowsy, nystagmus, blepharospasm	Pleocytosis, high protein	IV MP	Good
Goncalves et al. (18)	M/9	Hyperintense signal in mesial temporal lobes, periaqueductal region, lateral wall of ventricle and mammillary bodies and perivascular enhancement	Around 26 days	Altered mentality	Pleocytosis, high protein	IV Dex	Neurocogni-tive sequelae
Huang et al. (19)	M/18	Leptomeninges thickened and enhancement	Simultaneously	Headache	Pleocytosis, high protein	IV Dex, oral MP and hydroxychl-oroquine	Good

*Dex, dexamethasone; F, female; IG, immunoglobulin; Interval, interval between onset of lymphadenopathy and neurological manifestations; IV, intravenous; M, male; MP, methylprednisolone; PD, prednisolone*.

Byun et al. (8) found that the interval between the onset of lymphadenopathy and neurological symptoms was approximately 10–53 days. We noted an interval of 15 days to 3 months in our cases. CSF test results of tuberculous meningitis are similar to those of KFD (21). KFD may also exhibit symptoms of meningitis underscoring the importance of excluding bacterial and viral infections using CSF culture. T-spot examination and purified protein derivative also contribute to exclude tuberculosis infection. Guéguen et al. (21) found an increase in IFN-α levels in the CSF of patients with KFD without viral infection because of an upregulation of the IFN-α type I response. MRI findings, which include hyperintense T2 and FLAIR signal in the supratentorial white matter, deep gray matter, brain stem, cerebellum, temporal lobes, pons, and basal ganglia, can offer useful clues regarding CNS involvement (22). An excisional biopsy can provide a critical clue to the diagnosis.

Corticosteroids are the mainstay treatment for symptomatic KFD with encephalopathy. Cranial imaging after the initiation of corticosteroid therapy shows a reduction in the size and number of lesions (23). According to Rezai et al. (24), hydroxychloroquine has fewer adverse effects and is more efficacious than corticosteroids. The role of IVIG in KFD is for its anti-inflammatory effects for conditions where no autoantibody has been demonstrated (25). Immunosuppressive therapy has also been recommended for complicated cases with increased LDH and raised serum antinuclear antibody titers to prevent fatal outcomes (22). Excision of the affected lymph nodes may also be therapeutic (26). Glucocorticoids combined with IVIG can satisfactorily treat KFD complicated with hemophagocytic syndrome (27).

In conclusion, KFD is a rare and benign disease that should be considered in patients presenting with fever and lymphadenopathy. Patients with KFD can develop neurological symptoms, such as aseptic meningitis, multiple neuritis, or acute cerebellar ataxia. Conditions such as tuberculous meningitis, SLE, and infections should be excluded, and the possibility of encephalopathy-associated KFD should be considered. Therapy includes glucocorticoids combined with immunoglobulin. Antiviral therapy should be added if viral infections cannot be ruled out completely. Close follow-up is emphasized in these patients.

## Data Availability Statement

The original contributions presented in the study are included in the article/supplementary material, further inquiries can be directed to the corresponding authors.

## Ethics Statement

Written informed consent was obtained from the minor(s)' legal guardian/next of kin for the publication of any potentially identifiable images or data included in this article.

## Author Contributions

Y-TP and L-MC contributed equally to the manuscript and conceived, designed, drafted, wrote, and revised the manuscript. YX was responsible for literature search and data collection. Z-DF and H-GY critically revised the manuscript and provided expert feedback. All authors were involved in drafting the manuscript (or revising it), and all read and approved the final manuscript.

## Funding

This study was supported by National Natural Science Foundation of China (nos. 81202345, 81771762).

## Conflict of Interest

The authors declare that the research was conducted in the absence of any commercial or financial relationships that could be construed as a potential conflict of interest.

## Publisher's Note

All claims expressed in this article are solely those of the authors and do not necessarily represent those of their affiliated organizations, or those of the publisher, the editors and the reviewers. Any product that may be evaluated in this article, or claim that may be made by its manufacturer, is not guaranteed or endorsed by the publisher.

## References

[1] Kikuchi M. Lymphadenitis showing focal reticulum cell hyperplasia with nuclear debris and phagocytes: a clinicopathological study. Acta Haematol Jpn. (1972) 35:379–80.

[2] Fujimoto Y. Cervical subacute necrotizing lymphadenitis. A new clinicopathological entity Intern med. (1972) 20:920–27.

[3] Chong Y Kang CS. Causative agents of Kikuchi-Fujimoto disease (histiocytic necrotizing lymphadenitis): a meta-analysis. Int J Pediatr Otorhinolaryngol. (2014) 78:1890–7. 10.1016/j.ijporl.2014.08.019 25200851

[4] Kucukardali Y Solmazgul E Kunter E Oncul O Yildirim S Kaplan M. Kikuchi-Fujimoto disease: analysis of 244 cases. Clin Rheumatol. (2007) 26:50–4. 10.1007/s10067-006-0230-5 16538388

[5] Ramirez AL Johnson J Murr AH. Kikuchi-Fujimoto's disease: an easily misdiagnosed clinical entity. Otolaryngol Head Neck Surg. (2001) 125:651–3. 10.1067/mhn.2001.120431 11743471

[6] Cramer J Schmiedel S Alegre NG Schäfer H Burchard GD Merz H. Necrotizing lymphadenitis: Kikuchi–Fujimoto disease alias lupus lymphadenitis? Lupus. (2010) 19:89–92. 10.1177/0961203309345793 19933723

[7] Deaver D Horna P Cualing H Sokol L. Pathogenesis, diagnosis, and management of Kikuchi-Fujimoto disease. Cancer Control. (2014) 21:313–21. 10.1177/107327481402100407 25310212

[8] Byun JH Park SE Nam SO Kim YA Kim YM Yeon GM. Three children of meningoencephalitis with Kikuchi necrotizing lymphadenitis. Brain Dev. (2018) 40:251–5. 10.1016/j.braindev.2017.09.009 29050838

[9] Khishfe BF Krass LM Nordquist EK. Kikuchi disease presenting with aseptic meningitis. Am J Emerg Med. (2014) 32:1298.e1-2. 10.1016/j.ajem.2014.03.029 24746858

[10] Mahajan VK Sharma NL. Kikuchi-Fujimoto disease: immediate remission with ciprofloxacin. Int J Dermatol. (2004) 43:370–2. 10.1111/j.1365-4632.2004.01906.x 15117371

[11] Dumas G Prendki V Haroche J Amoura Z Cacoub P Galicier L. Kikuchi-Fujimoto disease: retrospective study of 91 cases and review of the literature. Medicine (Baltimore). (2014) 93:372–82. 10.1097/MD.0000000000000220 25500707 PMC4602439

[12] Selvanathan SN Suhumaran S Sahu VK Chong CY Tan NWH Thoon KC. Kikuchi-Fujimoto disease in children. J Paediatr Child Health. (2020) 56:389–93. 10.1111/jpc.14628 31576642

[13] Chiu CF Chow KC Lin TY Tsai MH Shih CM Chen LM. Virus infection in patients with histiocytic necrotizing lymphadenitis in Taiwan. Detection of Epstein-Barr virus, type I human T-cell lymphotropic virus, and parvovirus B19. Am J Clin Pathol. (2000) 113:774–81. 10.1309/1A6Y-YCKP-5AVF-QTYR 10874877

[14] Shapira Y Weinberger A Wysenbeek AJ. Lymphadenopathy in systemic lupus erythematosus. Prevalence and relation to disease manifestations. Clin Rheumatol. (1996) 15:335–8. 10.1007/BF02230354 8853165

[15] Quintás-Cardama A Fraga M Cozzi SN Caparrini A Maceiras F Forteza J. Fatal Kikuchi-Fujimoto disease: the lupus connection. Ann Hematol. (2003) 82:186–8. 10.1007/s00277-003-0611-7 12634955

[16] Sato Y Kuno H Oizumi K. Histiocytic necrotizing lymphadenitis (Kikuchi's disease) with aseptic meningitis. J Neurol Sci. (1999) 163:187–91. 10.1016/S0022-510X(99)00037-4 10371083

[17] Jasti DB Naveen Prasad SV Naveen T Vengamma B. Kikuchi-Fujimoto disease presenting as brainstem encephalitis with secondary blepharospasm. J Neurosci Rural Pract. (2016) 7:157–60. 10.4103/0976-3147.165395 26933369 PMC4750320

[18] Gonçalves LF Debelenko LV Bhambhani KJ Scheid A Altinok D. Histiocytic necrotizing lymphadenitis (Kikuchi-Fujimoto disease) with CNS involvement in a child. Pediatr Radiol. (2014) 44:234–8. 10.1007/s00247-013-2786-y 24091923

[19] Huang X Chen X Tong SW Wang Y Cai J Deng C. Kikuchi-Fujimoto disease complicated by aseptic meningitis and hemophagocytosis successfully treated with intrathecal dexamethasone. Heliyon. (2020) 6: e04193. 10.1016/j.heliyon.2020.e04193 32577568 PMC7305385

[20] Avkan-Oguz V Yapar N Ozakbas S Demir-Onder K Aktas E Alp-Cavus S. A case of fever of unknown origin: co-existence of Kikuchi-Fujimoto disease and acute disseminated encephalomyelitis (ADEM). Intern Med. (2010) 49:1823–6. 10.2169/internalmedicine.49.3633 20720367

[21] Guéguen A Sené T Maillart E Gout O. Encephalitis and CSF increased level of interferon-α in Kikuchi-Fujimoto disease. BMJ Case Rep. (2012) 2012:bcr0120125579. 10.1136/bcr.01.2012.5579 22927262 PMC3433502

[22] Choi YJ Lee SH Lee JK Nam TS Choi SM Kim BC. Aseptic meningitis in Kikuchi's disease mimicking tuberculous meningitis. Neurol Sci. (2013) 34:1481–3. 10.1007/s10072-012-1230-7 23124488

[23] Noursadeghi M Aqel N Pasvol G. Kikuchi's disease: a rare cause of meningitis? Clin Infect Dis. (2005) 41:e80-2. 10.1086/444563 16163623

[24] Rezai K Kuchipudi S Chundi V Ariga R Loew J Sha BE. Kikuchi-Fujimoto disease: hydroxychloroquine as a treatment. Clin Infect Dis. (2004) 39:e124–6. 10.1086/426144 15578393

[25] Altinel AE Sari E Sahin G Oguz MM Akçaboy M Zorlu P. Kikuchi-Fujimoto disease triggered by Salmonella enteritidis in a child with concurrent auto-immune thyroiditis and papilloedema. Paediatr Int Child Health. (2018) 38:298–301. 10.1080/20469047.2017.1420523 29307273

[26] Lee KY Yeon YH Lee BC. Kikuchi-Fujimoto disease with prolonged fever in children. Pediatrics. (2004) 114:e752–6. 10.1542/peds.2004-0485 15545615

[27] Kim YM Lee YJ Nam SO Park SE Kim JY Lee EY. Hemophagocytic syndrome associated with Kikuchi's disease. J Korean Med Sci. (2003) 18:592–4. 10.3346/jkms.2003.18.4.592 12923340 PMC3055072

